# The S-adenosylmethionine dependent *O*-methyltransferase PaMTH1: a longevity assurance factor protecting *Podospora anserina* against oxidative stress

**DOI:** 10.18632/aging.100029

**Published:** 2009-03-03

**Authors:** Birgit Kunstmann, Heinz D. Osiewacz

**Affiliations:** Department of Biological Sciences & Cluster of Excellence Macromolecular Complexes, Institute of Molecular Biosciences, Goethe University Frankfurt, 60438 Frankfurt am Main, Germany

**Keywords:** Podospora anserina, knock-out, reactive oxygen species, flavonoids, ageing, O-methyltransferase

## Abstract

PaMTH1 is an *O*-methyltransferase catalysing the methylation of vicinal hydroxyl groups of polyphenols. The protein accumulates during ageing of *Podospora anserina* in both the cytosol and in the mitochondrial matrix. The construction and characterisation of a *PaMth1* deletion strain provided additional evidence about the function of the protein in the protection against metal induced oxidative stress. Deletion of *PaMth1* was found to lead to a decreased resistance against exogenous oxidative stress and to a shortened lifespan suggesting a role of PaMTH1 as a longevity assurance factor in a new molecular pathway involved in lifespan control.

## Introduction

The cellular generation and accumulation of reactive oxygen species (ROS) and their potential to damage all types of biomolecules constitute the basis of the free radical theory of ageing [1,2]. In the past, numerous experimental studies revealed that elevated copper and iron levels in the cell are correlated with an enhanced formation of ROS [3-6]. The most hazardous among all ROS is the hydroxyl radical, the product of the reaction of H_2_O_2_ and the trace metals iron or copper during Fenton chemistry. Enhanced levels of intrinsic oxidative stress have been shown to induce a broad spectrum of oxidative damage of biomolecules including DNA, proteins and lipids [7]. Beside their role in the generation of ROS via Fenton-reaction, metals like copper or iron may lead to the transition of the naturally occurring antioxidants, for example flavonoids or vitamin c [8], to prooxidants which cause molecular damage of DNA, protein, or lipids. For example, in the presence of ferrous ions the plant flavonoid quercetin was reported to lead to increased oxidation of the glyceraldehyde 3-phosphate dehydrogenase (GAPDH) via the generation of the hydroxyl radical through the reaction of FeSO_4_ with quercetin [9].  Significantly, an age-dependent accumulation of metal ions in some model organisms, an enhanced ROS formation and a subsequent damage of biomolecules were shown in several studies [3,10]. Moreover, experimental data have been reported suggesting the acceleration of ROS-formation via the reaction of intrinsic flavonoid like substances or catechol derivatives with metals [11]. In this context it is important to note, that the flavonoid driven ROS-formation depends on the structural attributes of the flavonoid. Most of the polyphenols which in the absence of copper act as antioxidants can react with the metal via a vicinal dihydroxy system leading to the generation of ROS [11]. This reaction can be inhibited by substitution of the hydroxyl group via methylation and the formation of a methyl ester group. After such modifications the reaction with the metal ions is not possible and the prooxidant activities of the flavonoids are abolished [12]. *O*-methylation is performed by *O*-methyltransferases which are members of the S-adenosylmethionine (SAM)-dependent *O*-methyltransferase superfamily involved in the secondary metabolism of many species across all kingdoms.

Interestingly, in the filamentous fungus *Podospora anserina*, a well-established ageing model (for review: [13-15]), the accumulation of an *O*-methyltransferase (PaMTH1) was reported to accumulate in total and mitochondrial protein extracts during ageing [16-18]. PaMTH1 belongs to the cation-dependent subclass of SAM-dependent methyltransferases [19]. For instance, in animals and humans the endogenous substrate L-DOPA is methylated by the catechol-*O*-methyl-transferase (COMT), a class I *O*-methyltransferase which shows striking conservation with PaMTH1 [16]. Further analyses of the substrate specificity revealed that PaMTH1 uses flavonoids like quercetin and catechol derivates with vicinal dihydroxyl residues as substrates. More importantly, *PaMth1* over-expressing strains of *P. anserina* were found to display an increased resistance against metal induced oxidative stress and a significant prolonged lifespan in comparison to the wild-type strain s. These results strongly suggest a role of PaMTH1 in counteracting the age-related increase of ROS formation via the metal depending activation of phenolic compounds.

In the present study we report the generation and characterisation of a *PaMth1* deletion strain. This work complements previous studies about the analysis of the wild-type strain s, long-lived mutant grisea, and the recently generated strain over-expressing *PaMth1* [16-18] and strongly validates the protecting role of PaMTH1 and an impact of this enzyme on ageing and lifespan control.

## Results

**Figure 1.**
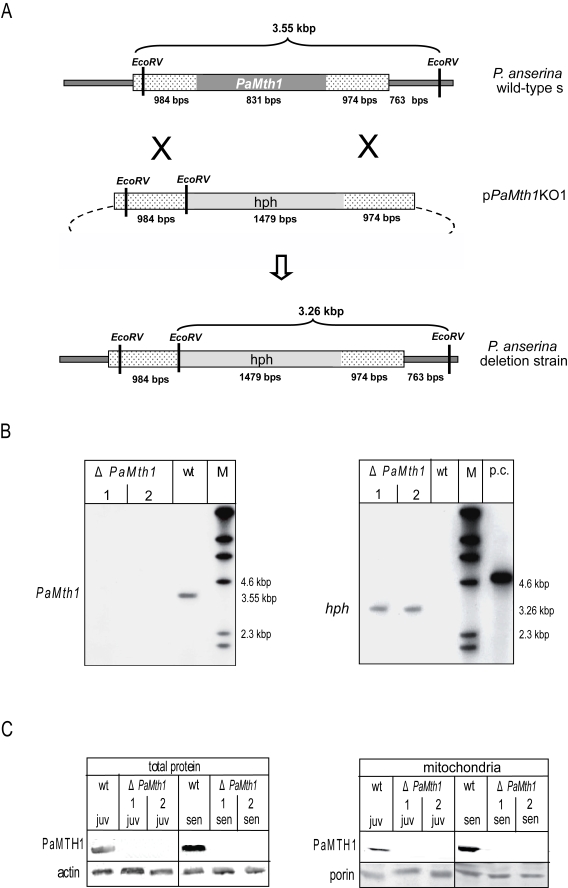
Construction and validation of a *PaMth1* deletion strain of *P. anserina*. (**A**) Physical maps and sizes of the restriction products for the genomic region bearing *PaMth1* and the recombined version with the hygromycin B resistance cassette. Reading frames of the two genes are indicated in grey. The genomic sequences flanking *PaMth1* are indicated by punctuation. Restriction sites of *Eco*RV are indicated. (**B**) Southern blot analysis of *Eco* RV digested wild-type strain s genomic DNA and genomic DNA of two secondary transformants isolated from a primary deletion strain. The *PaMth1* gene-specific probe (left panel) detects the 3.55 kbp fragment only in the sample of the wild-type s (wt) but not in the samples of the deletions strains (Δ*PaMth1*). The hygromycin B resistance gene-specific probe (right panel) detects the 3.26 kbp fragment only in the sample of the deletions strains. (**C**) Western blot analysis verifying the successful construction of a *PaMth1* deletion strain using total- and mitochondrial protein samples of juvenile and senescent *P. anserina* wild-type strain s and the secondary transformants of the deletion strain, respectively. The PaMTH1 specific antibody detects PaMTH1 in the samples of the wild-type s strains but not in samples of the deletion strains. As loading controls an actin specific antibody for total proteins and a porin specific antibody for the mitochondrial proteins were used.

In order to further elucidate the detoxifying role of PaMTH1 in *P. anserina* during ageing, we set out to construct a strain in which the *PaMth1* gene is replaced by a selectable marker gene.  Towards this goal a plasmid containing a hygromycin B resistance cassette and 5´ and 3´ flanking regions (aprox. 1.0 kbp) of the *PaMth1* gene was constructed and introduced into protoplasts of the *P. anserina *Δ*PaKu70 *strain (kindly provided by A. Sainsard-Chanet). Successful deletion of the *PaMth1* gene was verified in hygromycin B resistant transformants via Southern blot analyses using a *PaMth1*- and  hygromycin B (hph) specific probe, respectively  (Figure 1A, B). In the *Eco*RV digested genomic DNA of *P. anserina* wild-type strain s and in two secondary transformants of a deletion strain hybridised with a *PaMth1* specific probe a 3.5 kbp band is only detected in the sample of the wild-type strain s and not in the deletion strain. In genomic DNA of transformants a hygromycin B specific band is detectable which is absent from the genome of the wild-type s. These results demonstrate the correct integration of the resistance cassette into the *P. anserina* genome and the deletion of the *PaMth1* gene.

In order to verify the deletion of *PaMth1* at the protein level, total- and mitochondrial protein samples of juvenile and senescent strains of both the wild-type strain and of the selected transformants were analysed by Western blot experiments using a PaMTH1 specific polyclonal antibody. In the wild-type this antibody detected a protein in samples from juvenile and senescent cultures and verified the previously reported increase in abundance of PaMTH1 in senescent cultures (Figure 1C). In contrast, in none of the protein preparations from the two selected transformants a protein reacted with the PaMTH1 antibody clearly validating the successful construction of a *PaMth1* deletion strain.

**Figure 2.**
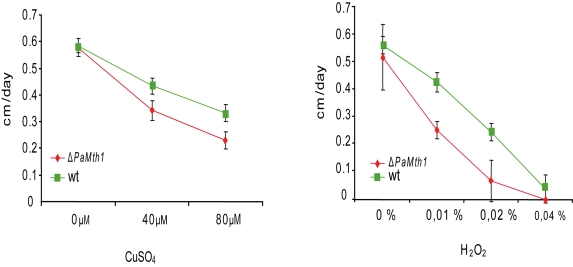
Growth rates of wild-type strain s and of the *PaMth1 *deletion strain on synthetic PASM medium containing different amounts of CuSO4 and of hydrogen peroxide, respectively. Pairs of strains (wild-type strain s and the *PaMth1* knock-out strain) were grown on one plate and growth rates were recorded over 3 days. Cultures of the *PaMth1* deletion strain (n = 20) were characterised by decreased growth rates compared to the wild-type strain s (n = 20) when incubated on medium containing 40 μM (p = 6.154e-08) or 80 μM (p = 1.921e-08) CuSO4 and 0.01% (p = 1.443e-08); 0.02% (p = 1.06e-07) or 0.04% (p = 0.99) hydrogen peroxide. Plates containing hydrogen peroxide were incubated in the dark.

**Figure 3.**
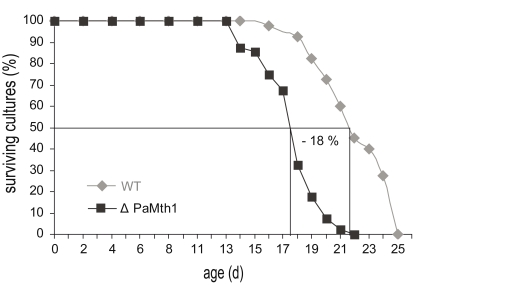
In comparison to wild-type strain s (n = 40), the mean lifespan of the PaMth1 deletion strain (n = 40) is decreased by 18%. Mean lifespan (wild-type strain s: 21 days and ΔPaMth1: 17 days) was determined on synthetic PASM medium.

In a previous study we demonstrated an increase in resistance against exogenous oxidative stress in *PaMth1* over-expression strains [18]. These strains display an improved growth rate on medium containing different concentrations of copper sulphate or hydrogen peroxide suggesting that *PaMth1* over-expression strains are characterised by reduced endogenous oxidative stress as the result of increased PaMTH1 activity during the whole lifespan [18]. In order to validate this idea, the growth rate of the *PaMth1* deletion strain was analysed on media containing or generating ROS.  For this purpose, juvenile isolates of the wild-type strain s and of a *PaMth1* deletion strain were inoculated on agar plates containing medium with different amounts of hydrogen peroxide and copper sulphate, respectively. On both media, the *PaMth1 *deletion strain displays a significantly decreased growth rate compared to the wild-type strain. Compared to the wild-type, the *PaMth1* deletion strain displays a significant decreased resistance against exogenous oxidative stress (Figure 2A, B) which may be the result of higher endogenous ROS levels in the deletion strain. Since a higher amount of oxidative stress leads to increased damage of all kinds of biomolecules and consequently may result in a shortened lifespan, the lifespan of the *PaMth1* deletion strain was determined. As expected, when compared to the wild-type, the deletion strain is characterised by an 18% shortened mean lifespan (Figure 3). These data are consistent with the results from the characterisation of the *PaMth1* over-expression strains which display a significant increase in lifespan [18].

The shortened lifespan and the decreased resistance against ROS raised the question of whether or not increased oxidative stress in the deletion strain can be monitored at the molecular level. To answer this question we investigated the abundance of carbonylated proteins in total- and mitochondrial protein samples of juvenile and senescent wild-type strain s and in deletion strains by Oxyblot analyses. Unexpectedly, the carbonyl content in the analysed samples in total and in the mitochondrial protein of the deletion strains does not significantly differ from the one of the wild-type strain (data not shown).

## Discussion

Recently, the *O*-methyltransferase PaMTH1 emerged as a new component of the complex network governing ageing and lifespan control in *P. anserina*. The constitutive over-expression of *PaMth1* resulted in a marked extension of  the lifespan  and  an  increased  resistance against ROS. The functional characterisation of the substrates of purified PaMTH1 protein revealed a specificity of this protein for flavonoids *in vitro *[18]. These flavonoids contain vicinal dihydroxyl residues which can react with metal ions and generate ROS. Thus, flavonoids are of potential danger since they may significantly add to the burden of endogenous ROS which increase during ageing of biological systems. Mechanisms counteracting the generation of ROS are therefore of important relevance. PaMTH1 as an *O*-methyltransferase able to enzymatically methylate vicinal dihydroxyl groups of flavonoids appears to be part of such pathways. In particular, as evidenced by different data suggesting an age-related increase of copper levels in the cytoplasm of *P. anserina* [20,21], it is reasonable to assume a role of PaMTH1 in protecting ageing cultures of the fungus against metal depending reactions of reactive flavonoid-like substrates. Unprotected these substrates would lead to ROS formation and subsequent molecular damage. In the current study we raised additional data complementing those from earlier investigations via the construction and characterisation of a *PaMth1* deletion strain.

For the generation of this strain we utilised the *P. anserina* strain Δ*PaKu70* carrying a deletion in a proteins necessary for non homologous end joining (NHEJ) [22]. In this strain homologous recombination of relative small flanking regions of the deletion target gene with a selectable marker gene on a plasmid is greatly facilitated. The correct integration of the selection marker and the deletion of the gene were shown by Southern blot analyses and validated on the protein level by a Western blot analysis with a specific PaMTH1 antibody.

The analyses of stress resistance on media supplemented with copper or hydrogen peroxide revealed a decreased growth rate of the deletion strain compared to the wild-type strain underlining the proposed detoxifying function of PaMTH1. In addition, more importantly, lifespan of the deletion strain is significantly shortened proposing again an important role of PaMTH1 in lifespan control of *P. anserina*. According to these results and to those from earlier investigations [18] it is very likely that PaMTH1 is involved in detoxification of its substrate(s) which otherwise react(s) with metals. If PaMTH1 is not available, as in the newly generated deletion strain, more unmethylated components with vicinal dihydroxy groups generating ROS and affecting the lifespan and resistance against ROS are available. However, surprisingly and in contrast to the analyses of the over-expressing strain showing a significant decrease in oxidised protein content, the carbonyl content in samples of the deletion strains displays no increase in the amount of these oxidised protein bands. The same amount of carbonylated proteins is found in the wild-type strain and in the deletion mutant. In this context it is important to note that also in wild-type cultures of different age no significant increase of overall carbonylation of proteins is observed in *P. anserina* [18]. It appears that not the overall carbonylation of proteins, but rather the specific carbonylation of sensitive but important proteins is of relevance for ageing. Such relatively small changes in the total carbonyl content cannot be detected by the applied Oxyblot analyses. A specific analysis (e.g. by mass-spectrometry) of proteins known as targets of ROS, like the mitochondrial aconitase, in different genetic backgrounds may help to clarify the impact of age-related protein damage in *P. anserina* and may provide new insights into complex molecular network involved in lifespan control and ageing in general.

## Methods

Strains, growth conditions and lifespan determination. In this study the wild-type strain s [23], the Δ*PaKu70* strain [22] and the short-lived Δ*PaMth1* strain were used. All strains were grown at 27°C on *P. anserina* synthetic medium (PASM) [24] under constant light. To obtain cultures of a defined age, mycelium from freshly germinated ascospores was placed on one side of a Petri dish containing PASM. Every 2-3 days, the growth front was marked. After reaching the other side of the Petri dish fresh plates of PASM were inoculated with a piece of the culture obtained from the growth front. Senescent cultures stopped growth and displayed hyper pigmentation. For further analysis, senescent cultures were obtained from plates shortly before growth arrest to inoculate fresh plates for further experiments (e.g. protein isolation).

Growth rate and lifespan determination. Lifespan and growth rate determination were performed with monokaryotic isolates from independent crosses. Freshly germinated spores were placed on race tubes with PASM. The time period of linear growth was recorded as lifespan in days. Growth rate was measured in  centimetres per  day.  Growth rates under oxidative stress conditions were recorded over 5 days starting with monokaryotic isolates from independent crosses of the wild-type strain s and the *PaMth1* deletion strains on PASM plates containing 40 μM and 80 μM CuSO4, respectively.

Alternatively, growth medium was supplemented by 0.01%, 0.02% and 0.04% hydrogen peroxide, respectively. To protect hydrogen peroxide from disintegration, plates were kept in the dark. For better comparability of growth rates, a wild-type strain s culture and a deletion strain were grown on the same plate.

Deletion of *PaMth1* in *P. anserina.* For deletion of the *PaMth1 *gene a vector was generated containing a hygromycin B resistance cassette for selection in *P. anserina* framed by approximately 1.0 kbps long 5' and 3' flanking sequences of *PaMth1.* These regions were amplified by PCR using sequence specific oligonucleotides introducing restriction sites for *Xho*I and *Hin*dIII (5'), and *Pst*I and *Xba*I (3'), respectively. After digestion of the plasmid pKO7 with the corresponding enzymes the two digested PCR products were cloned into this vector. The deletion vector pPaMth1KO1 was used to transform *P. anserina* protoplasts of the strain Δ*PaKu70*. The strains containing the recombined DNA were selected by hygromycin B resistance.

Transformation of *P. anserina.* Production, regeneration, and integrative transformation of *P. anserina* spheroplasts was performed as described [25, 26].

Isolation of mitochondria from *P. anserina.* Mitochondria were isolated from juvenile and senescent*P. anserina* cultures, respectively, according to a previously published protocol [27] with the following modifications. Crude mitochondria were isolated by differential centrifugation for 35 min at 15.000 g and 4° C. The mitochondrial pellet was resuspended in 1 ml of mitochondria isolation buffer (10 mM Tris, 1 mM EDTA, 0.33 M sucrose, pH 7.5) and layered on a 20-50% discontinuous sucrose gradient. After centri-fugation for 1 h at 100.000 g in a swing-out bucket rotor (TH 641) the mitochondria were banding between the 50 and the 36 % sucrose step. Approximately 30 ml mitochondrial isolation buffer without bovine serum albumin were added to the collected mitochondria fraction and centrifuged for 15 min at 15.000 g at 4 °C. For isolation of samples for Oxyblot analysis 100 mM DTT was added to the isolation buffer.

Oxy- and Western blot analyses. For Western blot analyses, total and mitochondrial protein samples (5-20 μg of protein) were boiled for 1 min in loading buffer [0.1 M Tris (pH 6.8), 6% SDS, 6% glycerol, 0.6 M β-mercaptoethanol] and were separated on a 12% SDS-PAGE using a Protean III unit (Bio-Rad, Hercules, CA, USA). Subsequently, proteins were transferred to a PVDF membrane (Millipore, Schwalbach, Germany) by using an electro-blotting device (Bio-Rad). Standard protocols were followed. Western blots were probed with a PaMTH1 rabbit polyclonal antibody (α-PaMTH1). Equal loading of total proteins was confirmed by incubation with a β-actin mouse antibody. The loading of mitochondria was controlled by incubation with an antibody against porin (*Neurospora crassa*). Detection of reacted proteins was performed by using IRDye 680 or 800 conjugated goat anti-rabbit or anti-mouse antibody and scanning the blots with an Odyssey infrared scanner (Li-Cor, Lincoln, NE, USA). For Oxyblot analyses, the total protein and mitochondrial protein samples were derivatised with di-nitro phenyl hydrazine using an Oxyblot kit (Intergene, Millipore, Schwalbach, Germany), separated in a 12% SDS PAGE and transferred onto a PVDF membrane (Immobilon). Blots were incubated with a rabbit anti-DNPH antibody and an IRDye 680 or 800 conjugated goat anti-rabbit. Reacted proteins were detected by scanning the blots with an Odyssey infrared scanner (Li-Cor).

## References

[1] Harman D (1956). A theory based on free radical and radiation chemistry. J Gerontol.

[2] Alvarez-Garcia O, Vega-Naredo I, Sierra V, Caballero B, Tomas-Zapico C, Camins A, Garcia JJ, Pallas M, Coto-Montes A (2006). Elevated oxidative stress in the brain of senescence-accelerated mice at 5 months of age. Biogerontology.

[3] Cook CI, Yu BP (1998). Iron accumulation in aging: modulation by dietary restriction. Mech Ageing Dev.

[4] Dunaief JL (2006). Iron induced oxidative damage as a potential factor in age-related macular degeneration: the Cogan Lecture. Invest Ophthalmol Vis Sci.

[5] Hofer T, Marzetti E, Xu J, Seo AY, Gulec S, Knutson MD, Leeuwenburgh C, Dupont-Versteegden EE (2008). Increased iron content and RNA oxidative damage in skeletal muscle with aging and disuse atrophy. Exp Gerontol.

[6] Ozcelik D, Uzun H (2009). Copper Intoxication; Antioxidant Defenses and Oxidative Damage in Rat Brain. Biol Trace Elem Res.

[7] Loft S, Hogh DP, Mikkelsen L, Risom L, Forchhammer L, Moller P (2008). Biomarkers of oxidative damage to DNA and repair. Biochem Soc Trans.

[8] Orhan H, Gurer-Orhan H, Vriese E, Vermeulen NP, Meerman JH (2006). Application of lipid peroxidation and protein oxidation biomarkers for oxidative damage in mammalian cells. A comparison with two fluorescent probes. Toxicol In Vitro.

[9] Schmalhausen EV, Zhlobek EB, Shalova IN, Firuzi O, Saso L, Muronetz VI (2007). Antioxidant and prooxidant effects of quercetin on glyceraldehyde-3-phosphate dehydrogenase. Food Chem Toxicol.

[10] Zatta P, Drago D, Zambenedetti P, Bolognin S, Nogara E, Peruffo A, Cozzi B (2008). Accumulation of copper and other metal ions, and metallothionein I/II expression in the bovine brain as a function of aging. J Chem Neuroanat.

[11] Jungbluth G (2000). Oxidation of flavonols with Cu (II), Fe (II) and Fe (III) in aqueous media. J Chem Soc, Perkin Trans.

[12] Zhu BT, Ezell EL, Liehr JG (1994). Catechol-O-methyltransferase-catalyzed rapid O-methylation of mutagenic flavonoids. Metabolic inactivation as a possible reason for their lack of carcinogenicity in vivo. J Biol Chem.

[13] Osiewacz HD, Kimpel E (1999). Mitochondrial-nuclear interactions and lifespan control in fungi. Exp Gerontol.

[14] Osiewacz HD (2002). Genes, mitochondria and aging in filamentous fungi. Ageing Res Rev.

[15] Scheckhuber CQ, Osiewacz HD (2008). Podospora anserina: a model organism to study mechanisms of healthy ageing. Mol Genet Genomics.

[16] Averbeck NB, Jensen ON, Mann M, Schägger H, Osiewacz HD (2000). Identification and characterization of PaMTH1, a putative o-methyltransferase accumulating during senescence of Podospora anserina cultures. Curr Genet.

[17] Groebe K, Krause F, Kunstmann B, Unterluggauer H, Reifschneider NH, Scheckhuber CQ, Sastri C, Stegmann W, Wozny W, Schwall GP, Poznanovic S, Dencher NA, Jansen-Dürr P, Osiewacz HD, Schrattenholz A (2007). Differential proteomic profiling of mitochondria from Podospora anserina, rat and human reveals distinct patterns of age-related oxidative changes. Exp Gerontol.

[18] Kunstmann B, Osiewacz HD (2008). Over-expression of an S-adenosylmethionine-dependent methyltransferase leads to an extended lifespan of Podospora anserina without impairments in vital functions. Aging Cell.

[19] Joshi CP, Chiang VL (1998). Conserved sequence motifs in plant S-adenosyl-L-methionine-dependent methyltransferases. Plant Mol Biol.

[20] Averbeck NB, Borghouts C, Hamann A, Specke V, Osiewacz HD (2001). Molecular control of copper homeostasis in filamentous fungi: increased expression of a metallothionein gene during aging of Podospora anserina. Mol Gen Genet.

[21] Borghouts C, Werner A, Elthon T, Osiewacz HD (2001). Copper-modulated gene expression and senescence in the filamentous fungus Podospora anserina. Mol Cell Biol.

[22] El-Khoury R, Sellem CH, Coppin E, Boivin A, Maas MF, Debuchy R, Sainsard-Chanet A (2008). Gene deletion and allelic replacement in the filamentous fungus Podospora anserina. Curr Genet.

[23] Rizet G (1953). Sur la longévité des souches de Podospora anserina. C R Acad Sci Paris.

[24] Hamann A, Brust D, Osiewacz HD (2007). Deletion of putative apoptosis factors leads to lifespan extension in the fungal ageing model Podospora anserina. Mol Microbiol.

[25] Osiewacz HD, Skaletz A, Esser K (1991). Integrative transformation of the ascomycete Podospora anserina: identification of the mating-type locus on chromosome VII of electrophoretically separated chromosomes. Appl Microbiol Biotechnol.

[26] Stumpferl SW, Stephan O, Osiewacz HD (2004). Impact of a disruption of a pathway delivering copper to mitochondria on Podospora anserina metabolism and life span. Eukaryotic Cell.

[27] Gredilla R, Grief J, Osiewacz HD (2006). Mitochondrial free radical generation and lifespan control in the fungal aging model Podospora anserina. Exp Gerontol.

